# Dataset on the behaviour of the Areolas da Estefania formation in Lisbon and its modelling using a state-dependent soil model

**DOI:** 10.1016/j.dib.2024.110391

**Published:** 2024-04-08

**Authors:** Antonio M.G. Pedro, David M.G. Taborda

**Affiliations:** a University of Coimbra, ISISE, ARISE, Department of Civil Engineering, Rua Luís Reis Santos – Pólo II, 3030-788 Coimbra, Portugal; b Department of Civil and Environmental Engineering, Imperial College London, Skempton Building, London SW7 2AZ, United Kingdom

**Keywords:** Geotechnical engineering, Granular media, Small strain stiffness, Soil strength

## Abstract

Experimental and computational data are presented for Areolas da Estefania, a geomaterial which is crucial for the development of the underground infrastructure of the city of Lisbon, Portugal. The experimental data comprise the particle size distribution of the material and measurements obtained during a series of strain-controlled triaxial compression tests performed on intact samples. The behaviour of this material at a wide range of strains, under constant mean effective stress levels of 130 kPa, 300 kPa and 400 kPa is established, with the presented dataset containing information on stress (mean effective stress and deviatoric stress) and strain states (axial strain and volumetric strain). These are complemented by the results of bender element tests imposing vertically-travelling waves for characterisation at very small strains. Complementarily, the computational dataset establishes a reference reproduction of the response of Areolas da Estefania using a material model which combines a non-linear small stiffness formulation with a state-dependent strength and plastic dilatancy. Overall, this dataset can be used as a reference when assessing the behaviour of other samples of Areolas da Estefania or comparable materials, or when evaluating constitutive models for granular geomaterials.

Specifications Table

**Table:** 

Subject	Geotechnical Engineering and Engineering Geology
Specific subject area	Characterisation of the mechanical behaviour of granular soils under a wide range of strains
Data format	Raw, Analyzed
Type of data	Table
Data collection	Samples of Areolas da Estefania were extracted from Lisbon, Portugal, and, after physical characterisation, were subjected to strain-controlled drained shearing under constant mean effective stress in a triaxial cell of the Bishop and Wesley type. Axially-mounted local instrumentation was employed to characterise the mechanical response at small strains. This was complemented by bender element tests on additional samples imposing vertically-propagating shear waves. Numerical modelling of the observed behaviour was performed with the material model IC MAGE M02 combining a small-strain stiffness formulation with state-dependent strength and dilatancy.
Data source location	City: Lisbon Country: Portugal
Data accessibility	1. Experimental data Repository name: Zenodo Data identification number: 10.5281/zenodo.10888728 Direct URL to data: https://zenodo.org/records/10888728 2. Modelling data Repository name: Zenodo Data identification number: 10.5281/zenodo.10452912 Direct URL to data: https://zenodo.org/records/10452912

## 1 Value of the Data

• Despite its importance in the context of building new underground infrastructure in Lisbon, Portugal, there is a lack of experimental data on the mechanical response of the Areolas da Estefania formation, preventing the calibration of the constitutive models required for accurate soil-structure interaction analyses. This dataset characterises the stiffness and strength of this material under a wide range of strains and mean effective stress levels.
• The experimental data provides a reference against which results of new tests on samples collected from Areolas da Estefania formation can be compared for assessing reliability of experimental procedures and interpretation of observed behaviour.
• The behaviour of intact samples of natural materials contained in this dataset presents a considerably sterner test to the capabilities of constitutive models for geomaterials. The experimental data includes all information required to develop, calibrate or evaluate material models for natural soils.
• The modelling dataset illustrates the strengths and shortcomings of a practice-oriented constitutive model for soils which can be used as a benchmark for alternative calibrations of the same model or evaluation of other material models.

## 2 Background

The construction of a complex shaft designed to improve access to one of the largest stations of the Lisbon underground railway system required advanced numerical modelling of the associated soil-structure interaction problem to be performed. The accuracy of such analyses is intrinsically dependent on the capabilities of the constitutive model used in the numerical simulations and on the quality of the experimental data used in its calibration. To this end, samples from the Areolas da Estefania formation were collected from the vicinity of the future location of the shaft to perform an advanced characterisation of the mechanical response of this material. This consisted of bender element tests, as well as of drained triaxial compression tests under constant mean effective stress levels. The resulting experimental data is complemented by a dataset consisting of computational simulations using a state-dependent soil model which can be readily used in simulations of complex geotechnical structures in this material.

## 3 Data Description

The structure and contents of the experimental and modelling datasets presented in this paper are outlined in Table 1, Table 2, respectively. The experimental dataset [1] is divided into three *.xlsx files. The first of these files describes the particle size distribution of samples collected at relevant depths. The second file contains the results of two bender element tests provided in separated worksheets, each containing the values of the normalized mean effective stress ($p^{\prime }/p_{ref}^{\prime }$) and the corresponding shear modulus (G) obtained. A value of 100kPa was adopted for the reference mean effective stress, $p_{ref}^{\prime }$. The third file includes the results of three triaxial tests performed under constant mean effective stress levels of 130 kPa, 300 kPa and 400 kPa. For each test, the measured values of axial strain ($\varepsilon_{ax}$), volumetric strain ($\varepsilon_{vol}$), mean effective stress ($p^{\prime }$) and deviatoric stress (q) are provided. Additionally, the generalized deviatoric strain ($E_{d}$), calculated for triaxial stress states using Eq. (1), and the tangent shear modulus ($G_{tan}$), obtained using Eq. (2), are also included.

(1) $$E_{d}=\sqrt{3}\cdot \left(\varepsilon_{ax}-\frac{\varepsilon_{vol}}{3}\right)$$

(2) $$G_{tan}=\frac{\Delta q}{3\cdot \Delta \varepsilon_{ax}-\Delta \varepsilon_{vol}}$$

**Table 1:** Outline of the experimental dataset [1].

File	Type of test	Test designation	Variables
AE-Data-PSD.xlsx	Particle size distribution	PSD-AE-08 PSD-AE-18 PSD-AE-21	Particle size (mm) Percentage passing (%)
AE-Data-BenderElements.xlsx	Bender element	BE-AE-1 BE-AE-2	Sample photographs Sample characteristics $p^{\prime }/p_{ref}^{\prime }$ G(MPa)
AE-Data-Triaxial.xlsx	Triaxial	T-AE-130 T-AE-300 T-AE-400	Sample photographs Sample characteristics $\varepsilon_{ax}\;\left(\%\right)$ $\varepsilon_{vol}\;\left(\%\right)$ $p^{\prime }\;\left(kPa\right)$ q(kPa) $E_{d}\;\left(\%\right)$ $G_{tan}\;\left(MPa\right)$

**Table 2:** Outline of the modelling dataset [2].

File	Worksheet	Contents
AE-Model-Stiffness.xlsx	M-AE-Gmax	Modelled elastic stiffness at small strains $p^{\prime }/p_{ref}^{\prime }$ – G(kPa)
	M-AE-EdG-I130	Modelled stiffness variation with strain for test T-AE-130 $E_{d}\;\left(\%\right)-G_{tan}\;\left(kPa\right)$
	M-AE-EdG-I300	Modelled stiffness variation with strain for test T-AE-300 $E_{d}\;\left(\%\right)-G_{tan}\;\left(kPa\right)$
	M-AE-EdG-I400	Modelled stiffness variation with strain for test T-AE-400 $E_{d}\;\left(\%\right)-G_{tan}\;\left(kPa\right)$
M-AE-I130.csv		Modelled behaviour for test T-AE-130 containing lists of: $\varepsilon_{ax}\;\left(\%\right);\;\varepsilon_{vol}\;\left(\%\right);\;p^{\prime }\;\left(kPa\right);\;q\;\left(kPa\right);\;e\;\left(\;\right);\;$$E_{d}\;\left(\%\right);\;G_{tan}\;\left(kPa\right)$
M-AE-I300.csv		Modelled behaviour for test T-AE-300 containing lists of: $\varepsilon_{ax}\;\left(\%\right);\;\varepsilon_{vol}\;\left(\%\right);\;p^{\prime }\;\left(kPa\right);\;q\;\left(kPa\right);\;e\;\left(\;\right);\;$$E_{d}\;\left(\%\right);\;G_{tan}\;\left(kPa\right)$
M-AE-I400.csv		Modelled behaviour for test T-AE-400 containing lists of: $\varepsilon_{ax}\;\left(\%\right);\;\varepsilon_{vol}\;\left(\%\right);\;p^{\prime }\;\left(kPa\right);\;q\;\left(kPa\right);\;e\;\left(\;\right);\;$$E_{d}\;\left(\%\right);\;G_{tan}\;\left(kPa\right)$
AE-Calibration.xlsm	Gmax	Calibration record for determining parameters $G_{ref}$ and $m_{G}$ based on results of bender element tests (Figure 2(a))
	G-Reduction	Calibration record for determining parameters $a_{1},\;a_{2}$ and b based on results of triaxial tests (Figure 2(b))

The modelling dataset [2], as shown in Table 2, consists of the simulated response for the three triaxial tests contained in the experimental dataset using the IC MAGE M02 model [3,4] with the parameters listed in Table 3. The *.csv files correspond to the output of a Python script [5] which integrates the model equations for the specific case of drained triaxial compression under constant mean effective stress levels. The results of these simulations are compared to the experimental data in Fig. 3. Additionally, the file AE-Model-Stiffness.xlsx includes further details on the modelled response in terms of the small-strain stiffness properties of the material, covering both stiffness at very small strains (compared against the experimental results from bender element testing in Fig. 2(a)) and the variation of shear modulus with strain level (illustrated together with the corresponding experimental data in Fig. 2(b)). A final file, AE-Calibration.xlsm, records the results of the calibration process associated with the component of the model responsible for simulating the small-strain stiffness response of the model. By assisting in its calibration, this part of the modelling dataset facilitates the use of the same constitutive model for other materials.

**Table 3:** Material parameters for Areolas da Estefania using IC MAGE M02 model [3,4].

Parameter	Value		Parameter	Value
*Elastic part*			*Plastic part*	
$G_{ref}\;\left(kPa\right)$	72,450.0		$M_{CS}$	1.42
$p_{ref}\;\left(kPa\right)$	100.0		$k_{1}$	4.01
$m_{G}\;\left(-\right)$	0.51		$k_{2}$	0.0
$a_{0}\;\left(-\right)$	$1.0\times 10^{-6}$		$l_{1}$	6.23
$a_{1}\;\left(-\right)$	$1.31\times 10^{-5}$		$l_{2}$	0.0
$\;a_{2}\;\left(-\right)$	1.84		$e_{CS,ref}$	0.675
b(−)	0.60		λ	0.0
$R_{G.min}\;\left(-\right)$	0.01		ξ	0.0
ν(−)	0.2			

## 4 Experimental Design, Materials and Methods

**Fig. 1:**
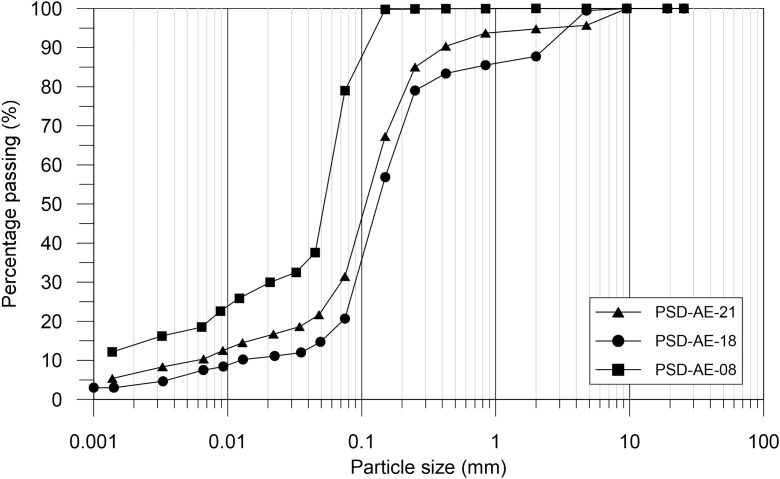
Particle size distribution for samples collected at depths of 8 m, 18 m and 21 m.

The samples tested were extracted from two boreholes performed in the backyard of the Quintão building, Lisbon, Portugal [6,7] at depths of 8 m, 18 m and 21 m. At the indicated depths, a 76 mm-diameter thin-walled sampler with a PVC liner was used to retrieve intact samples. In order to characterize the particle size distribution, traditional sieving and sedimentation tests as set out in the standard BS 1377-2 [8] were undertaken. Despite some natural variability, all samples classify as sandy materials with fines (Fig. 1), predominantly composed of quartz minerals (≈60%) followed by feldspar (≈15%) [6]. The experimental programme comprised different types of tests that were specifically chosen in order to characterize in detail the stress-strain behaviour of the Areolas da Estefânia formation. The tests were performed using a stress-path cell apparatus Bishop and Wesley type, which allows the automatic control of the vertical, radial and backpressure stresses applied to the sample. Throughout the tests the strains in the samples were measured using local and external high-resolution instrumentation. The sample characteristics, initial conditions and photographs of the samples before and after each test are presented in the experimental dataset [1]. Prior to testing, all samples were first saturated to a minimum B value of 0.98. In the triaxial tests, the consolidation stress (mean effective stress) adopted was in agreement with estimated in situ stress level. As a result, the samples, which were retrieved at depths of 8 m, 18 m and 21 m, were consolidated to isotropic stresses of 130 kPa, 300 kPa and 400 kPa, respectively. To remove the effect of mean effective stress level from the small-strain stiffness behaviour of each sample, the triaxial compression tests were performed under constant $p^{\prime }$. Under such loading conditions, the stiffness at very small strains $G_{max}$ (Eq. 3) is assumed to remain constant since changes in void ratio are negligible up to intermediate strain levels. This allows a simpler normalization of the behaviour in terms of $G_{tan}/G_{max}$ (Eq. 4), which is then solely a function of a measure of shear strain. This stress-path was imposed by increasing the vertical stress in the sample, while simultaneously reducing the horizontal stress. During the entire test the drainage line of the sample was kept open, and a slow rate of stress variation was imposed, thus ensuring that all samples were sheared under drained conditions.

To execute the bender element tests, the pedestal and top cap of the triaxial apparatus were replaced with equivalent pieces that incorporate the bender elements. In the top extremity of the sample a shear-wave with known input signal, a single sine pulse, was transmitted through the sample. The receiver, located at the bottom of the sample, recorded the output signal, allowing the determination of the arrival time and, consequently, of the maximum shear modulus. To define the arrival time of the vertically-propagating and horizontally-oscillating shear wave, the ‘first arrival’ method from the time-domain framework was applied, as discussed in [9]. To evaluate the influence of the mean effective stress on the shear stiffness at very small strains, each sample was isotopically consolidated for different stress levels, with the bender element test being performed for each of those levels.

**Fig. 2:**
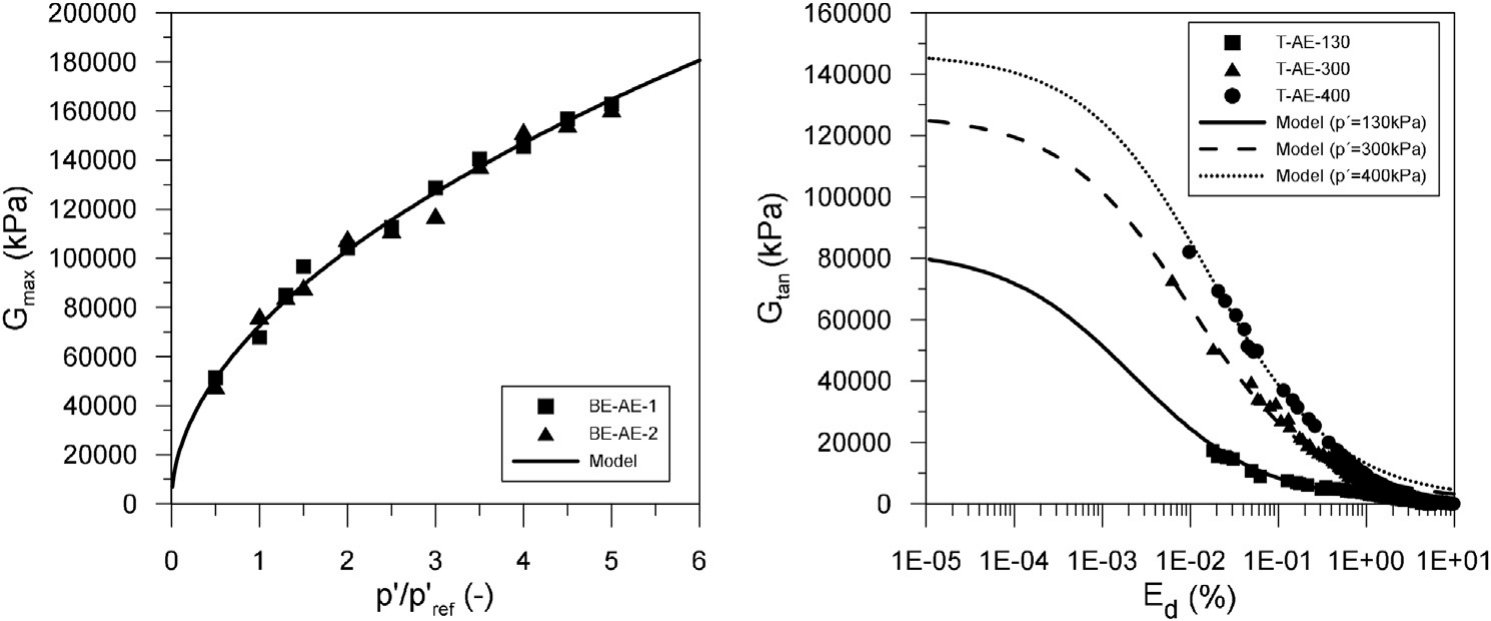
Experimental and modelling datasets for Areolas da Estefania in terms of (a) maximum stiffness obtained using bender element testing and (b) reduction of stiffness with strain level observed in triaxial testing.

The calibration of IC MAGE M02 [3,4] is outlined in detail in [3] and therefore only a brief description of the various steps required are provided herein, with particular emphasis given to those where modifications are required to address specific challenges resulting from the characteristics of the studied material. The first aspect of the model to be calibrated is that determining the shear stiffness at very small strains ($G_{max}$), which is obtained directly from the Bender Elements tests by fitting a power law:

(3) $$G_{max}=G_{ref}\cdot {\left(\frac{p^{\prime }}{p_{ref}^{\prime }}\right)}^{m_{G}}$$

where $p_{ref}^{\prime }\;$is a reference pressure (adopted as 100 kPa), $G_{ref}$ is the shear modulus obtained in bender element testing at a mean effective stress of $p_{ref}^{\prime }$ and $m_{G}$ is a parameter determining the non-linearity of the relationship between $G_{max}$ and the mean effective stress $p^{\prime }$. The outcome of this fitting process is shown in Fig. 2(a). The IC MAGE M02 model allows for the variation of $G_{ref}$ with void ratio according to a wide range of empirical expressions [3,4]. However, the obtained data suggested a very limited effect of the void ratio, possibly due to the inherent variability of natural samples (see Fig. 1). As a result, a single value of $G_{ref}$ is used, although any new test performed on this material should evaluate this aspect independently.

The expression governing the reduction of shear stiffness with strain level is [10]:

(4) $$\frac{G_{tan}}{G_{max}}=R_{G,min}+\frac{1-R_{G,min}}{1+{\left(\frac{E_{d}}{a^{*}}\right)}^{b}}$$

where $G_{max}$ is determined using Eq. (3) for the same value of mean effective stress as that applied when $G_{tan}$ is calculated, $E_{d}$ denotes the generalized deviatoric strain (i.e. the second invariant of the strain tensor, see Eq. (5)), $a^{*}$ is the deviatoric strain at which the shear modulus has reduced to $G_{max}\cdot \left(1+R_{G,min}\right)/2$ (Eq. (6)), b controls the non-linearity of the shear stiffness reduction and $R_{G,min}$ is the elastic shear stiffness at very large strains (here assumed to take a value of 1% to avoid very small values of $G_{tan}$).

(5) $$E_{d}=\sqrt{\frac{4}{6}\left({\left(\varepsilon_{xx}-\varepsilon_{yy}\right)}^{2}+{\left(\varepsilon_{yy}-\varepsilon_{zz}\right)}^{2}+{\left(\varepsilon_{zz}-\varepsilon_{xx}\right)}^{2}\right)+\gamma_{xy}^{2}+\gamma_{yz}^{2}+\gamma_{zx}^{2}}$$

(6) $$a^{*}=a_{0}+a_{1}\cdot {\left(p^{\prime }/p_{ref}^{\prime }\right)}^{a_{2}}$$

Assuming $a_{0}=1.0\times 10^{-6}$ to prevent unrealistically low values of the reduction parameter $a^{*}$, a least-square method approach was employed to fit the remaining parameters simultaneously ($a_{1}$, $a_{2}$ and b) to the $E_{d}-G_{tan}$ data from the three triaxial tests, yielding $a_{1}=1.23\times 10^{-5}$, $a_{2}=1.89$ and b=0.60. The type of tests executed – triaxial compression under constant mean effective stress – facilitates this calibration since, for these loading conditions, $G_{max}$ is assumed to remain constant and thus does not require updating (see Eq. (3)). Fig. 2(b) illustrates both the experimental and the numerical datasets for the stiffness of Areolas da Estefania for a wide range of strains. A Poisson's ratio of ν=0.2 is assumed for this material.

**Fig. 3:**
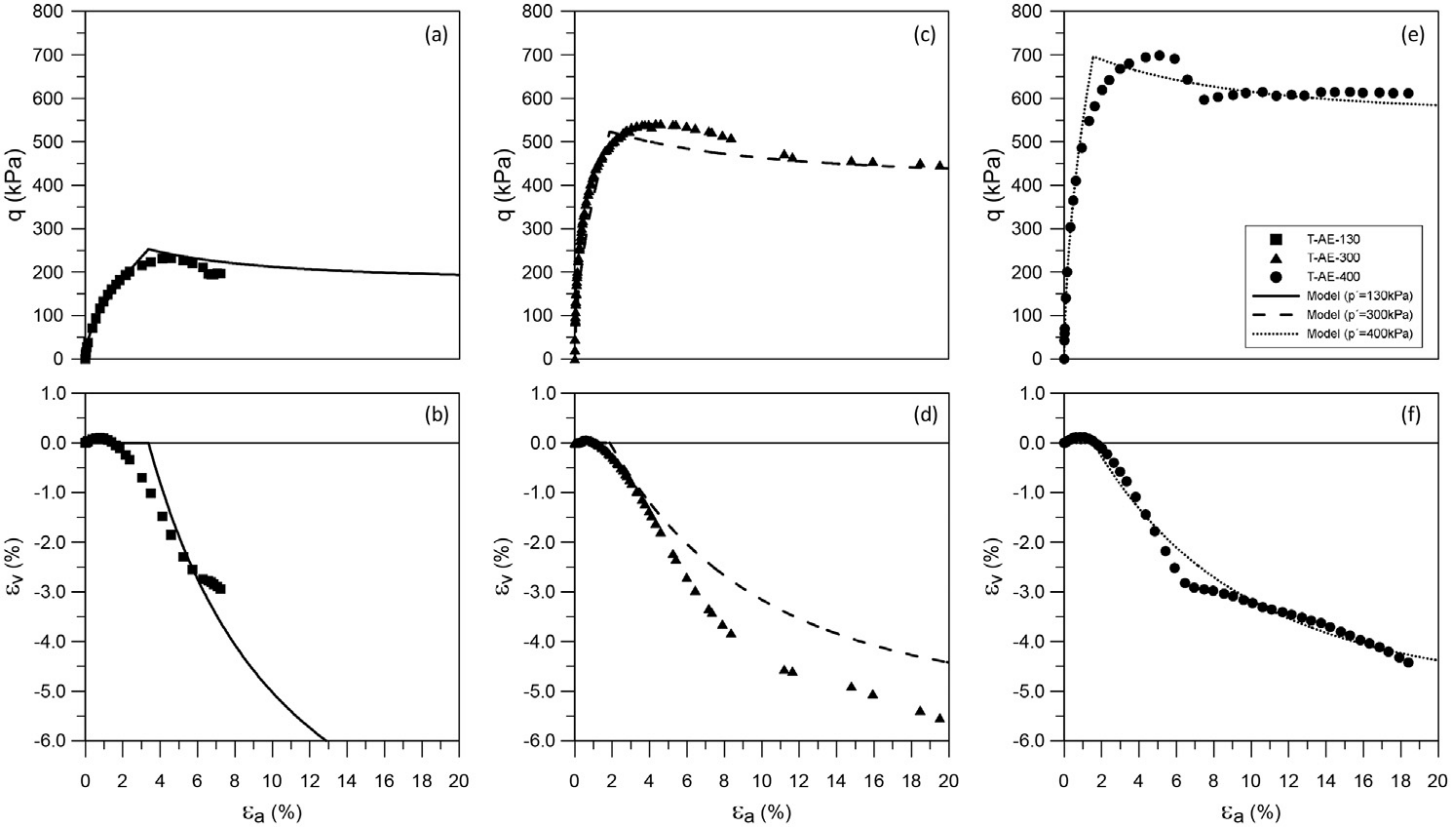
Experimental and modelling datasets in terms of $\varepsilon_{ax}-q\;$and $\varepsilon_{ax}-\varepsilon_{vol}$ spaces for triaxial compression test (a,b) T-AE-130, (c,d) T-AE-300 and (e,f) T-AE-400 of Areolas da Estefania.

The plastic part of the constitutive model governs yielding and hardening/softening associated with the shearing of the material. At its core, IC MAGE M02 (see [3] for details) is a critical state-based soil model, though its formulation can be adapted for situations where the clear identification of critical state is difficult (an interesting discussion on this issue is presented in [3]). Herein, one such adaption is proposed: rather than a Critical State Line defined using a power law, a constant value of void ratio (i.e. independent of mean effective stress, obtained by adopting λ=ξ=0.0) is chosen as a “dilatancy limit” based on the final volumetric strain ($\varepsilon_{vol,f}$) interpreted from the triaxial compression test data. This quantity can be converted to a final void ratio ($e_{f}$) using:

(7) $$e_{f}=e_{0}-\left(1+e_{0}\right)\cdot \varepsilon_{vol,f}$$

where $e_{0}$ is the initial void ratio. The estimated values of $\varepsilon_{vol,f}$ are listed in Table 4, together with the associated values of $e_{f}$. The dilatancy limit was placed at a void ratio $e_{CS,ref}=e_{f}=0.675$, which corresponds to the average of the values obtained for test T-AE-300 and T-AE-400, which were similar and considerably larger than that for T-AE-130. The parameter controlling the peak strength, $k_{1}$, can be approximated, for $k_{2}=0.0$, using $k_{1}\approx \left(M_{s,\;peak}-M_{CS}\right)/\psi_{0}^{*}$, where $\psi_{0}^{*}=e_{0}-e_{CS,ref}$ is the initial value of the state parameter, $M_{s,peak}$ is the peak value of the stress ratio $q/p^{\prime }$ and $M_{CS}$ is the stress ratio corresponding to the angle of shearing resistance at very large strains (adopted as $35^{\circ }$, as suggested in [6,7]). The obtained values are listed in Table 4, with an average value of 4.01 being adopted in this calibration. Similarly, the parameter controlling the plastic potential, $l_{1}$, can be estimated for $l_{2}=0.0$ using $l_{1}\approx M_{d,peak}/\psi_{0}^{*}$, with $M_{d,peak}$ being the peak value of the dilatancy rate, measured as $M_{d}=\Delta \varepsilon_{vol}/\Delta \varepsilon_{d}$, where $\varepsilon_{d}=2/3\cdot \left(\varepsilon_{ax}-\varepsilon_{rad}\right)$. For the present dataset, an average value $l_{1}=6.23$ was determined. The final set of parameters is listed in Table 3 and the modelled response, using [5], is compared to that measured in the laboratory in Fig. 3.

**Table 4:** Interpretation of the main characteristics of the plastic response of the tested samples.

Test	$e_{0}$	$\varepsilon_{vol,f}$	$e_{f}$	$\psi_{0}^{*}$	$M_{s,peak}$	$k_{1}$	$M_{d,peak}$	$l_{1}$
T-AE-130	0.544	3.5	0.598	−0.106	1.81	3.00	0.62	4.75
T-AE-300	0.594	6.0	0.690	−0.056	1.81	4.85	0.55	6.86
T-AE-400	0.595	4.0	0.659	−0.055	1.75	4.19	0.57	7.07
Adopted			0.675			4.01		6.23

## Limitations

While the experimental dataset provides a valuable contribution to the characterisation of the mechanical response of an important formation within the geological and geotechnical context of Lisbon, Portugal, it includes a reduced number of samples. This is, at least in part, a consequence of the complex process required to extract intact samples of geomaterials, which limits the number of tests that can be carried out. Moreover, the limited data available also impacts the modelling of the behaviour of the material, hindering the objective calibration of specific aspects of the adopted material model. In the present case, the effect of void ratio on the simulated stiffness and strength of Areolas da Estefania could not be observed conclusively. This resulted in simplifications being introduced to the hypoelastic formulation and to the yield and plastic potential functions employed in IC MAGE M02, which could limit the application of the determined parameter set to samples of similar physical properties. While further testing on this material could help clarify these aspects of the model, it is also true that the variability of natural samples is unlikely to allow a perfect match of the observed behaviour.

## Ethics Statement

The authors follow the ethical requirements for publication in Data in Brief and confirm that the current work does not involve human subjects, animal experiments, or any data collected from social media platforms.

## CRediT authorship contribution statement

**Antonio M.G. Pedro:** Conceptualization, Data curation, Investigation, Methodology, Writing – review & editing. **David M.G. Taborda:** Conceptualization, Methodology, Software, Validation, Writing – original draft, Writing – review & editing.

## Data Availability

IC MAGE M02 modelled behaviour for intact samples of Areolas da Estefania (Original data) (Zenodo). Experimental characterisation of intact samples of Areolas da Estefania (Original data) (Zenodo).
